# Type-specific Distribution of Cervical hrHPV Infection and the Association with Cytological and Histological Results in a Large Population-based Cervical Cancer Screening Program: Baseline and 3-year Longitudinal Data

**DOI:** 10.7150/jca.48357

**Published:** 2020-08-25

**Authors:** Fangbin Song, Hui Du, Aimin Xiao, Chun Wang, Xia Huang, Zhihong Liu, Meifang Zhao, Hongjian Men, Ruifang Wu

**Affiliations:** 1 Department of Obstetrics and Gynecology, Peking University Shenzhen Hospital, Shenzhen 518036, Guangdong, PR China; 2 Shenzhen Key Laboratory on Technology for Early Diagnosis of Major Gynecological diseases, Shenzhen 518036, Guangdong, PR China; 3 Buji Street Family Planning Service Center, Buji Street, Longgang District, Shenzhen 518129, PR China

**Keywords:** Human papillomavirus, genotype, cytology, cervical cancer screening, vaccination

## Abstract

**Objectives:** This study aimed to describe the study design, and to analyze the type-specific distribution of cervical high-risk human papillomavirus (hrHPV) infection and its association with cytological and histological results in a large population-based screening program in Buji Street, Shenzhen, China.

**Methods:** A total of 10,186 women aged 21-70 years were co-tested by Cobas4800 HPV assay and liquid-based cytology. Women were referred to colposcopy by virtue of being HPV16/18-positive, Other hrHPV-positive/ cytology ≥ASCUS, or HPV-negative/ cytology ≥LSIL. Three-year histological follow-up data were recorded.

**Results:** The overall prevalence of hrHPV infection was 11.1%; among them, the highest type was Other hrHPV (8.9%), followed by HPV16 (1.6%) and HPV18 (0.6%). Moreover, the prevalence of hrHPV and that of HPV16 increased with cytological severity (*P_trend_* <0.001). In the baseline phase, 106 women had cervical intraepithelial neoplasia 2/3 (CIN2/3) and six had cervical cancers. During 3-year follow-up, 12 cases of CIN2/3 and no cancers were identified. For HPV16-positive women with normal cytology, the baseline risks of CIN2/3 or worse (CIN2+/CIN3+) were 15.5% (7.0-23.9%) and 4.2% (1.4-8.5%) respectively. For Other hrHPV-positive women with normal cytology, the cumulative 3-year risks of CIN2+/CIN3+ were 3.1% (1.0-5.2%) and 0.7% (0.3-2.1%) respectively. Strikingly, 75.8% (322/425) of abnormal cytology and 50.9% (29/57) HSIL cytology were attributed to Other hrHPV infection in HPV-positive women. Similarly, Other hrHPV infection led to large proportions of CIN2 (62.7%) and CIN3+ (43.9%) over 3-year follow-up.

**Conclusions:** The co-testing modality is a feasible, effective and safe option for cervical cancer screening in urban population. Great importance should also be attached to 'genotypes excluding HPV16/18' and separate detection of each genotype when considering screening and vaccination strategy.

## Introduction

Cervical cancer is a common gynecologic oncology and a global public health problem 1. Over the past several decades, cytology-based screening programs have significantly reduced the burden of cervical cancer 2. In spite of the inherent advantages of cytology in cervical screening, cytology suffers in a relatively low sensitivity 3. Moreover, the positive predictive value (PPV) of cytology for cervical intraepithelial neoplasia 2 or worse (CIN2+) decreased among vaccinated women 4. Persistent high-risk human papillomavirus (hrHPV) infection has become well recognized as the main cause of cervical cancer 5, 6. This leads to the development of HPV tests for hrHPV detection as a screening method and HPV vaccine for cervical cancer prevention and control.

HPV testing has been incorporated in cervical cancer screening to triage cases with atypical cells of undetermined significance (ASCUS) cytology 7, or as a co-testing with cytology to maximize the detection rate of cervical precancers in women aged 30 years and older 8. Importantly, adding hrHPV testing to screening can improve the effectiveness and safety of screening over routine cytology 9. In addition, partial genotyping (HPV16/18) was proved sensitive for detecting cervical precancers in all cytological categories, and was particularly valuable in risk evaluation for women with ASCUS and low-grade squamous intraepithelial lesion (LSIL) 10. Besides, the utility of these types has also been recommended in risk-based management 7, 11. However, more detailed evidences on the risk stratification for cervical abnormality by genotypes are needed. Prophylactic vaccination represents another efficient preventive strategy against cervical cancer 12, and the effectiveness of HPV vaccines depends on the prevalence of HPV genotypes among populations in different geographical regions as well as the proportion of cervical lesions covered by vaccines 12.

Recently, we conducted a prospective, population-based cervical cancer screening program in Buji Street, Longgang District, Shenzhen, China (Southern China). Buji Street is located in the middle of Shenzhen city, where has a rapid economic development, resulting in Shenzhen's high-mobility and dense population, but relatively backward medical care. No screening program had ever been organized in Buji Street, and the vast majority of the people had never been screened for cervical cancer, particularly via HPV testing. Given the limited data on cervical HPV infection and correlative cytology or histology findings, it's hard to establish effective cervical cancer prevention strategies.

From this large cohort, we provide the study design, describe the prevalence of hrHPV genotypes, and analyze the association between cervical hrHPV genotypes and cytological abnormalities at baseline as well as histological results through 3-year follow-up. We hope this large-scale population-based study is instructive for the prevention of cervical cancer in urban areas similar to Buji street, Shenzhen, China.

## Materials and Methods

### Study population and design

**Figure 1:**
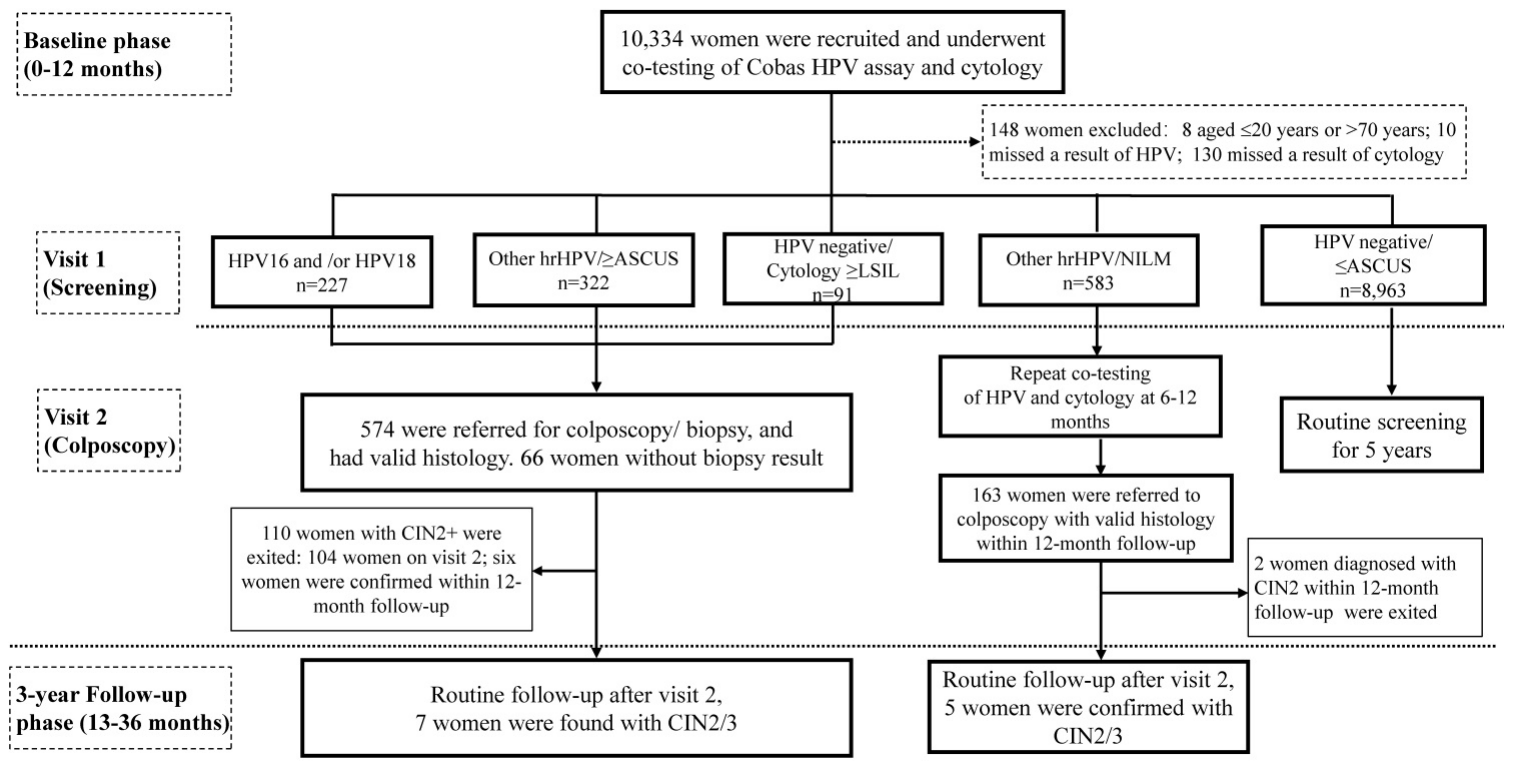
Flow chart and study design. Abbreviations: ASCUS, atypical squamous cells of undetermined significance; CIN, cervical intraepithelial neoplasia; hrHPV, high-risk human papillomavirus; LSIL, low-grade squamous intraepithelial lesion; NILM, negative for intraepithelial lesions or malignancy.

This prospective observational study was conducted in two phases: a baseline phase, and a three-year longitudinal phase (Figure 1). Women attending for cervical cancer screening program in Buji Street, Shenzhen, China were screened from Aug 2016 to Nov 2019. The participants were local permanent residents, mainly the aborigines of Shenzhen. Inclusion criteria were as follows: aged 21-70 years, nonpregnant, had a sexual history, without hysterectomy or radiotherapy. Women who had a history of cervical precancers or cancers, or who showed symptoms of invasive cervical cancers, or who attending for follow-up for previously abnormal screening results were excluded. 10,334 participants were recruited and cotested by liquid-based cytology and HPV assay at either Buji Street or the gynecological clinic in Peking University Shenzhen Hospital (PUSH). The study was conformed to the 2013 Declaration of Helsinki and approved by the PUSH Ethics Committee (No. PUSHGYN2015005). All the specimens and information registration were obtained with written informed consent from the participants before enrollment. The privacy of the participants was fully insured during or after data collection. Data from the baseline phase and the follow-up phase are reported here (Figure 1).

### Primary screening and cytology/HPV test (Visit 1)

After written informed consent was given, a brief medical history was acquired. Routine gynecological examination was performed by PUSH gynecologists, and the specimens of cervical exfoliated cells were brushed at the same time. Cervical exfoliated cell samples were obtained via a standardized sampling procedure by removing cervical secretions and rotating brush 3 times. Afterwards, the specimen on the clinician-sampling brush was placed in a small bottle containing 12mL of SurePath^®^ Preservative Solution (TriPath Imaging, Inc) and centralized to PUSH for cytology/HPV tests. 1.5mL SurePath specimen was prepared for HPV assay on a Cobas4800 system (Roche, USA) per the instruction of manufacturer. The residual specimen was prepared for AutoCyte^®^ thin-layer liquid-based cytology test (TriPath Imaging, Inc). Cytology slides were reviewed by the senior cytologists from PUSH according to the Bethesda classification system (TBS) 2014 13. The liquid-based cytology (LBC) diagnoses were classified into negative for intraepithelial lesion or malignancy (NILM), ASCUS, LSIL, atypical squamous cells cannot exclude high-grade lesion (ASC-H), high-grade squamous intraepithelial lesion (HSIL), and atypical glandular cells (AGC). A threshold of ≥ASCUS was used to define abnormal cytology. The results of HPV testing were divided into four hierarchical categories: HPV16+, HPV18+ (negative for HPV16, positive for HPV18, with or without 12 other types), Other hrHPV (negative for HPV16/18 and positive for any of 12 other types), and negative.

### Colposcopy and histological diagnoses (Visit 2)

Women met any of the results (1) HPV16 and/or HPV18 positivity or (2) Other hrHPV positivity and cytology ≥ASCUS or (3) HPV negative and cytology ≥LSIL were called back for colposcopy-directed biopsy according to the Preventive Oncology International (POI) microbiopsy protocol 14. Histological diagnoses were according to the colposcopy-directed biopsy or, if classified worse, on the histology result of the specimen excised by loop electrosurgical excision procedure (LEEP), conization, or hysterectomy. High-grade lesions were based on H&E stains in conjunction with p16 immunohistochemistry slides. The histological outcomes were reviewed by PUSH pathologists blinded to results of other tests, and classified as negative, LSIL (CIN1), HSIL (Including CIN2/CIN3), and cancers. Adenocarcinoma in situ (AIS) was included into CIN3. Patients with histological diagnosis of CIN2+ exited the study and received an appropriate management according to the diagnosis and treatment procedures of PUSH. If the patient fails to meet the requirements of clinical colposcopy and lacks histological results, the outcome should be determined comprehensively according to the results of HPV/cytology test, and the occurrence of HPV-negative/ (NILM or ASCUS) during the follow-up was treated as histologic normal and suggested to routine screening.

### Follow-up phase

Women had positive screening results (hrHPV-positive or LBC ≥LSIL) and without a histology of CIN2+ in the baseline phase were eligible for the three-year histological follow-up phase (Figure 1). Follow-up methods including telephone callbacks and outpatient visits for reexamination at PUSH, with questionnaires/data registries being completed simultaneously. Examinations with collection of a cervical exfoliated cell specimen for both cytology and HPV testing were annually followed up. Women exited the study once diagnosed with CIN2+, while those who weren't will continue in the follow-up phase. To optimize disease ascertainment at the end of the follow-up phase (Nov 2019), all nonpregnant women eligible for the follow-up phase were called back and offered an exit colposcopy-directed biopsy/ endocervical curettage. Among patients who had more than one histological results, the most abnormal diagnosis was accumulated.

### Statistical analysis

Since women with a result of Other hrHPV positivity/ NILM cytology did not meet the criteria for either immediate colposcopy or return to routine screening and would be deferred to 6-12 months follow-up (Figure 1), to minimize the possibility of verification bias being introduced by missing and/or delayed diagnoses at enrollment, histological results detected within 0-12 months after baseline tests were allocated to the baseline analysis, whereas histological results obtained at a later time (up to 36 months after baseline tests) were allocated to the '3-year' longitudinal analysis (Figure 1) 15. Distribution of each hrHPV genotypes at baseline by histological grades were calculated. The Mantel-Haenszel chi-square test was carried out to investigate any linear trend in hrHPV prevalence and age groups, or cytological abnormalities, or histological results, as well as HPV16/18 positivity. The absolute risks (ARs) for CIN2+/CIN3+ were determined for each combination of HPV types and cytology categories, and the respective 95% confidence intervals (CIs) for ARs were estimated by bootstrapping (1,000 times) 16. Analyses were carried out using SPSS software (IBM Corporation, version 24.0). All analyses were two-sided, and *P*-value <0.05 was considered statistically significant.

## Results

### Characteristics of the study population

Of the 10,334 participants, 8 women aged ≤20 years or >70 years and 140 women missed cytology or HPV results were excluded. Eventually, a total of 10,186 women, aged from 21 to 70 years were enrolled in the analysis. The mean age was 40.7 years (±7.8 years). Among them, 9,469 (93.0%) were considered NILM, 717 (7.0%) were diagnosed as abnormal cytology, 421 (4.1%) were classified as ASCUS, 29 (0.3%) were affected by AGC, 158 (1.6%) were diagnosed as LSIL, 47 (0.5%) were categorized as ASC-H, and 62 (0.6%) were positive for HSIL (Table 1). In the baseline, 9,462 (97.4%) were diagnosed as normal pathology, 137 (1.4%) as CIN1, 61 (0.6%) as CIN2, 45 (0.5%) as CIN3, and 6 (0.1%) cases as cervical cancers. The follow-up ranges from 0 to 35 months, during the 3-year follow-up period, 12 cases had subsequently diagnosed as CIN2+ (6 cases of CIN2 and 6 cases of CIN3), no case of invasive cervical cancer or AIS was detected. Therefore, through cumulative 3-year follow-up, 9,570 (97.3%) were diagnosed as normal pathology, 146 (1.5%) as CIN1, 67 (0.7%) as CIN2, 51 (0.5%) as CIN3, and 6 (0.1%) cases as cervical cancers (Table 1).

### Distribution of different hrHPV infection patterns and stratified by age groups

The overall prevalence of hrHPV infection was 11.1%, and the highest type was Other hrHPV (8.9%), followed by HPV16 (1.6%) and HPV18 (0.6%) (Table 2). The enrolled women were categorized according to age into 4 groups, with 559 (5.5%), 4,330 (42.5%), 3,894 (38.2%), and 1,403 (13.8%) women aged <30, 30 to 39, 40 to 49, and ≥50 years, respectively. The age-specific prevalence of HPV infection was showed in Table 2. The HPV positivity rate had two age peaks, ≥50 (12.7%) and <30 (12.0%) years. While the highest rate of HPV16 positivity was among women aged <30 years (2.3%), followed by women aged ≥50 years (1.9%).

### Prevalence of hrHPV infection according to cytology categories

The overall hrHPV positivity rate among women with abnormal cytology was 59.3% (425 of 717), which was approximately 7.4 times greater than that among women with normal cytology (7.5%, 707 of 9,469). The prevalence of hrHPV increased with cytological severity, from 7.5% among women with NILM to 91.9% among those with HSIL (P_trend_<0.0001), similar results were showed for HPV16 positivity and HPV16 and/or HPV18 positivity (P_trend_<0.0001, Table 3). Prevalence of each combination of HPV and cytology categories was showed in Table 3. The prevalence of HPV-positive/normal cytology results was 6.9% (707 of 10,186), which was more than 1.5 times that of HPV-positive/ abnormal cytology results (4.2%, 425 of 10,186). Moreover, 5.6% (9 of 161) of HPV16 infection led to ASC-H, and 15.5% (25 of 161) led to HSIL; while 2.3% (21 of 905) of Other hrHPV infection contributed to ASC-H, and 3.2% (29 of 905) contributed to HSIL, indicating that HPV16 infection was associated with more-severe cytology results (ASC-H: χ^2^=4.9, p =0.026; HSIL: χ^2^=36.1, p <0.0001) than Other hrHPV infection. However, among HPV-positive women, 75.8% (322/425) of abnormal cytology and 50.9% (29/57) HSIL cytology were attributed to Other hrHPV infection.

### Association of HPV test result with histology stratified by age

Table 4 shows the prevalence of hrHPV infection according to cumulative 3-year histological diagnosis and age groups. The prevalence of hrHPV infection detected in ≤CIN1, CIN2, and CIN3+ were 7.0%, 98.5%, and 94.7% respectively, and positively associated with histological severity (P_trend_ <0.0001). Similar results were showed for HPV16 positivity and HPV16 and/or HPV18 positivity (P_trend_ <0.0001). For women with histological results of ≤CIN1 and CIN2, the most prevalent infection type was Other hrHPV (5.4% and 62.7%, respectively); for women diagnosed as CIN3+, the most frequent genotype was HPV16 (47.4%), followed by Other hrHPV (43.9%). Of the 12 cases diagnosed as CIN2+ during 3-year follow-up phase, all of them were infected with Other hrHPV at baseline. Hierarchical typing results grouped as HPV16, HPV18, Other hrHPV, and negative showed a significant linear association with histological severity (P_trend_ <0.0001 overall and stratified by age groups, Table 4).

### Association of HPV test result with histology stratified by cytology categories

Table 5 shows the distribution of HPV types and cumulative 3-year histological results stratified by cytology categories. Hierarchical typing results grouped as HPV16, HPV18, Other hrHPV, and negative showed a significant linear association with histological severity stratified by cytology categories except for LSIL (LSIL, P_trend_=0.216; Other cytology categories, P_trend_ <0.001, Table 5). Notably, there was no high-grade lesion found at baseline or 3-year follow-up among women negative of HPV infection but with a LSIL cytology.

### The baseline and cumulative 3-year risks of each co-testing result for CIN2+/CIN3+

Crude risks of each co-testing (HPV type/cytology) results for CIN2+/CIN3+ detected at baseline and at cumulative baseline plus 3-year follow-up were estimated in Table 6. Of women with a result of HPV16 positivity/ NILM cytology, the baseline risks for CIN2+/CIN3+ were 15.5% (7.0-23.9%) and 4.2% (1.4-8.5%), respectively. However, of women with a result of Other hrHPV positivity/ NILM cytology, the baseline risk for CIN2+ was 1.2% (0.6-3.7%), and the cumulative 3-year risk for CIN2+/CIN3+ increased to 3.1% (1.0-5.2%) and 0.7% (0.3-2.1%) respectively. Of women positive for HPV16/ASCUS, the baseline risks for CIN2+/CIN3+ were 25.0% (12.5-40.6%) and 9.4% (3.1-19.4%), respectively. Of women positive for Other hrHPV/ASCUS, the baseline risks for CIN2+/CIN3+ were 7.4% (3.7-11.7%) and 0.6% (0.6-1.8%) respectively, and the cumulative 3-year risks for CIN2+/CIN3+ increased to 8.4% (4.2-12.7%) and 1.8% (0.6-4.2%) respectively (Table 6).

## Discussion

The distribution and infection patterns of HPV types in various areas provides a scientific basis for the establishment of HPV-based screening strategies and vaccination programs 17. Our results showed that the overall prevalence of hrHPV infection was 11.1%, which was higher than 7.9% in a recent study conducted in Central China 17 and similar with 10% in Addressing the Need for Advanced HPV Diagnostics (ATHENA) trial conducted in American by performing Cobas4800 HPV assay 3. Whereas the overall prevalence of HPV16/18 was 1.6%, lower than that of 1.95% in Central China 17. In addition, the hrHPV positive rate was highest in women aged ≥50 years possibly due to the declined immune ability and the weakened ability in eliminating infections in older women, and followed by women younger than 30 years who appear to sexually active.

The issue whether extending genotyping and individually detecting 'Other hrHPV' or not is still under debate and of increasing importance 18, 19. One study considers that identification of other 12 genotypes as a pool could provide sufficient information for screening 18. In another study, however, Cuzick et al. showed that HPV16 retains its high risk, while HPV31 and especially HPV33 emerge as important types with higher positive predictive values (PPVs) than HPV18 19. Thus, individual HPV genotypes within commonly grouped categories of “Other hrHPV types” do not carry equal risk, and separate assessment of HPV 33 and 31 needs to be reconsidered 19, 20. In this cohort, Other hrHPV infection was most prevalent, accounting for 79.9% of hrHPV infections, and contributed to substantial cytological abnormalities as well as large proportions of CIN2/CIN3 (62.7% and 43.9%, respectively) over 3-year follow-up. With the advent of the vaccine era, prevalence of HPV16/18 infections and related CIN2+ rate are expected to decline, both in vaccinated women and unvaccinated women due to herd protection 21, 22. Furthermore, the eradication of HPV16/18 could lead to a relative increase in prevalence of Other hrHPV types and related CIN2+ rate 23. Along with these changes, cervical cancer screening and vaccine strategies will face challenges 24.

To fit the change, policies should be adjusted to focus more attention on extended genotypes excluding HPV16/18, which is useful for the development of tailored HPV vaccines targeting Other hrHPV genotypes and for the adaption of screening algorithms for increasingly vaccinated cohorts. Moreover, the current HPV assays may require a modification, such as adding more previously low prevalence hrHPV subtypes and separating each genotype 25. Recently, a growing number of low-cost HPV tests have been developed to distinguish extended genotypes 20, 26, which makes the better understanding of individual types and the better evaluation of vaccine effects. In order to reduce the burden of cervical diseases, the bivalent vaccine (covering HPV16 and 18), quadrivalent vaccine (covering HPV 16, 18, 6 and 11) and nonvalent vaccine were licensed in China successively since 2016. However, from the results of our study, the bivalent and quadrivalent vaccine were unable to sufficiently cover the common HPV types, or to provide sufficient protection for cervical precancer or cancer. Fortunately, nonvalent vaccine might be more suitable for this population.

Detailed information on prevalence of each combination of HPV/cytology results was also described. The prevalence of HPV-positive/normal cytology result was reported ranged from 1.9% to 9.8% (Here was 7.5%) 10, 18, 27. This is one of the most common positive screening results encountered in daily practice 28 and more than twice that of HPV-positive/abnormal cytology results (Here was 3.6%) 7. The HPV-positive women with normal cytology should be followed by either repeated co-testing within 12 months or immediate colposcopy for positive genotyping of HPV16 alone (11.5%, 81/707) or HPV16/18 (17.5%, 124/707) according to the guidelines 7, which reduces the burden of follow-up and mental stress of women involved. Herein, the guideline strategy would defer colposcopy for 51.5% (583 of 1,132) of HPV-positive women. Moreover, only 7 (2.4%) cases of CIN2 and 2 (0.7%) cases of CIN3 were detected among women with a result of Other hrHPV positivity/NILM cytology through 3-year follow-up. Therefore, findings from the current study supported the guidelines above 7, and provided a basis for the establishment of regional cervical cancer screening programs. Moving forward, consistent with results from prior studies 10, 27, HPV prevalence increased with the aggravation of cytological grades. Additionally, HPV16 infection was more likely associated with more-severe cytological abnormality than Other hrHPV infection.

In addition to assessing results of HPV and/or cytology, it is valuable to know the risk for high-grade lesions conferred by specific genotypes when evaluating screening strategies. The 2019 ASCCP guidelines have moved from result-based management to risk-based management, and recommend that women having ≥4% risk of CIN3+ immediately of their positive screening test are referred for colposcopy and potential biopsy, whereas those having risk below that threshold require retesting within 1 year 11. In view of these facts, risks of each combination of HPV types and cytological categories for histologically high-grade lesions were estimated in this study. Attention was focused on women with a negative cytology, the absolute risks of HPV16 positive/normal cytology for CIN2+/CIN3+ at baseline were up to 15.5% (7.0-23.9%) and 4.2% (1.4-8.5%) respectively. In the ATHENA trial, women with HPV16 infection and normal cytology at baseline had a cross-sectional risk of CIN3+ two to three times that of the colposcopy referral threshold, and after 3 years this risk was five-fold higher 29, 30. Although HPV18 showed a lower risk than Other hrHPV, HPV18 is of great importance given its significant association with cancers and difficult-to-detect diseases particularly glandular lesions in the endocervical canal 6, 31. Therefore, based on the results of the current study and prior studies, women positive for HPV16/18 even with normal cytology are suggested to be referred for colposcopy. Notably, of women with a result of Other hrHPV positivity/ NILM cytology, the baseline risk for CIN2+ was only 1.2% (0.6-3.7%), which was low enough for precancer/cancer to permit follow-up in 6-12 months retesting with the expectation of viral clearance, thus avoiding immediate colposcopy.

In this program, women with a result of HPV negative/ LSIL cytology were referred to immediate colposcopy. However, there was no high-grade lesion found in this subgroup through 3-year follow-up. A prior study documented that risk for follow-up high-grade lesions in this subgroup was low and also that no cervical cancer was diagnosed, thus repeating co-testing after one year was an appropriate option for these women 32. Therefore, women with a result of HPV negative/LSIL cytology might not be referred for colposcopy immediately, and repeated co-testing within 12 months might be the suitable management, which is in line with the guidelines 11. In addition, there were three cases of CIN3 and one case of CIN2 missed by HPV test; one likely explanation is that the technical sensitivity of HPV assay, which may be affected by some factors such as cervicitis and vaginitis, is a limiting factor cannot be excluded in the misdetection of some cases; another is that the occurrence of the disease may be attributed to viral DNA integration into the host genome and transformation of cervical epithelial cells to precancerous lesions 33. Nevertheless, HPV/cytology co-testing remains the safest screening methods in the urban population.

Although cervical cancer screening guidelines have recommended management measures for women with each positive HPV/cytology co-testing results based on the principle of “equal management of equal risks''^34^, there still lack data particularly on risk of each HPV genotype/ cytology grade in Southern China to verify the effectiveness, and this prospective study from an large urban population fills this gap by analyzing the prevalence of hrHPV infection, and its association with cervical abnormalities. A comprehensive characterization of the infection status quo will provide both the Chinese government and public with the information needed to tailor screening strategies and interventions. Herein, most CIN2+ lesions (112 cases) were diagnosed earlier in the baseline, resulting in a reduced detection of CIN2+ lesions (12 cases) in subsequent rounds, which was consistent with a recent study conducted in Hongkong, China 8. The slow process of cervical oncogenesis and POI microbiopsy protocol conducted at baseline, which minimized the rate of missed diagnosis, may also contribute to the low number of CIN2+ in 3-year follow-up. Since only a small number of high-grade lesions and no cancers were found during follow-up after the baseline phase, the screening model of HPV/cytology co-testing followed by three colposcopy referral requirements conducted in this program is feasible, effective, and safe in urban populations.

There are several limitations of this study. Firstly, there was no records of using vaccination, occupations, birth control, smoking history, and number of sexual partners in the baseline data collected; despite that, most of the women enrolled in the population may be unvaccinated against HPV given that HPV vaccines were approved in mainland China since 2016, and have not been incorporated to National Immunization Program yet, thus the coverage is low to nonexistent. In addition, there might be some verification bias since not all the women underwent biopsy, for instance, some women with a result of HPV negative/ (NILM or ASCUS) were referred to routine screening, and some women infected with Other hrHPV not producing a detectable cytological abnormality (≥ASCUS) would be deferred to 6-12 months follow-up. However, cumulative histological results through 1-year or 3-year follow-up were included in the analysis.

An important strength of this study lies in the large-scale, and well-organization of the screening program, as well as the standard screening procedures, such as the standardized sampling process, the uniform requirements for colposcopy referral, and the standard POI protocol for colposcopy/biopsy. Another strength is the stratification of the association between HPV genotypes and histological grades by age groups and cytology status. Moreover, comprehensive and complete data, ranging from HPV prevalence and cytology outcome to histological follow-up results, were provided in this large prospective study, which provides necessary information for the clinical management of different subgroups of women based on HPV and cytology results. Furthermore, we also tried our best to obtain the 3-year follow-up data, due to the high mobility and dense population in highly urbanized Buji Street, it is arduous and essential to obtain such longitudinal data.

Based on a large cohort of women with cytology/HPV results and follow-up histology, this study described a screening modality of hrHPV/cytology co-testing in urban China, and explored the potential role of hrHPV testing and genotyping in risk assessment by different cytological categories, providing necessary information for cervical screening strategies and a new insight in assessing the potential efficacy of regional vaccinations. Our data support the guidelines using genotyping for HPV16/18 as a triage test since its high risk for cervical diseases. Moreover, our results highlight the importance of 'hrHPV genotypes excluding HPV16/18' and separate detection of these genotypes similar to HPV16/18 when considering triage and vaccination strategy given its considerably high prevalence and significant contribution to a high proportion of cervical abnormalities. The screening model of HPV/cytology co-testing is suitable for urban areas similar to Buji street, Shenzhen, China.

## Figures and Tables

**Table 1:** Characteristics of eligible women

Characteristics	Eligible women, N (%)
Age: Mean ± SD	40.7±7.8
Cytology (baseline)	10,186
NILM	9,469 (93.0)
ASCUS	421 (4.1)
LSIL	158 (1.6)
AGC	29 (0.3)
ASC-H	47 (0.5)
HSIL Abnormal (≥ASCUS)	62 (0.6) 717 (7.0)
Histology (baseline)	9,711
Normal	9,462 (97.4)
CIN1	137 (1.4)
CIN2	61 (0.6)
CIN3	45 (0.5)
Cancers	6 (0.1)
Histology (cumulative 3-year)	9,840
Normal	9,570 (97.3)
CIN1	146 (1.5)
CIN2	67 (0.7)
CIN3	51 (0.5)
Cancers	6 (0.1)

Abbreviations: AGC, atypical glandular cells; ASC-H, atypical squamous cells, cannot exclude high-grade lesion; ASCUS, atypical squamous cells of undetermined significance; CIN, cervical intraepithelial neoplasia; HSIL, high-grade squamous intraepithelial lesion; LSIL, low-grade squamous intraepithelial lesion; NILM, negative for intraepithelial lesion or malignancy.

**Table 2:** hrHPV infection patterns and stratified by age groups

HPV status	Age: Mean ± SD	Total	Age group N (%)			
			<30	30-39	40-49	≥50
**hrHPV test**	40.7±7.8	10,186	559	4,330	3,894	1,403
hrHPV negative	40.7±7.7	9,054 (88.9)	492 (88.0)	3,862 (89.2)	3,475 (89.3)	1,225 (87.3)
hrHPV positive	41.1±8.2	1,132 (11.1)	67 (12.0)	468 (10.8)	419 (10.7)	178 (12.7)
**hrHPV infection types**						
**HPV16 positive**	39.7±8.6	161 (1.6)	13 (2.4)	80 (1.8)	42 (1.1)	26 (1.9)
HPV16 single infection	39.7±8.5	119 (1.2)	6 (1.1)	65 (1.5)	29 (0.7)	19 (1.4)
HPV16 multiple infection ^a^	39.6±9.1	42 (0.4)	7 (1.3)	15 (0.3)	13 (0.3)	7 (0.5)
**HPV18 positive**	41.5±8.2	66 (0.6)	2 (0.4)	28 (0.6)	23 (0.6)	13 (0.9)
HPV18 single infection	42.1±7.6	46 (0.5)	1 (0.2)	17 (0.4)	20 (0.5)	8 (0.6)
HPV18 multiple infection ^b^	40.1±9.5	20 (0.2)	1 (0.2)	11 (0.3)	3 (0.1)	5 (0.4)
**Other hrHPV positive**	41.3±8.2	905 (8.9)	52 (9.3)	360 (8.3)	354 (9.1)	139 (9.9)

Abbreviations: SD, standard deviation. ^a^ HPV16 multiple infection indicates HPV16+/HPV18+, or HPV16+/ HPV18+/Other hrHPV+, or HPV16+/Other hrHPV+; ^b^ HPV18 multiple infection indicates HPV18+/Other hrHPV+.

**Table 3:** Association of hrHPV infection with cytology category

HPV Status	Cytology category						Cytology
	NILM	ASCUS	LSIL	ASC-H	HSIL	AGC	≥ASCUS
**hrHPV test**							
hrHPV negative	8,762 (92.5)	201 (47.7)	50 (31.6)	16 (34.0)	5 (8.1)	20 (69.0)	292 (40.7)
hrHPV positive	707 (7.5)	220 (52.3)	108 (68.3)	31 (66.0)	57 (91.9)	9 (31.0)	425 (59.3)
**hrHPV infection types**							
HPV16/18	124 (1.3)	46 (10.9)	16 (10.1)	10 (21.3)	28 (45.2)	3 (10.3)	103 (14.4)
HPV16 positive	81 (0.9)	32 (7.6)	12 (7.6)	9 (19.1)	25 (40.3)	2 (6.9)	80 (11.2)
HPV16 single infection	62 (0.7)	21 (5.0)	9 (5.7)	5 (10.6)	20 (32.3)	2 (6.9)	57 (7.9)
HPV16 multiple infection ^a^	19 (0.2)	11 (2.6)	3 (1.9)	4 (8.5)	5 (8.1)	0 (0.0)	23 (3.2)
HPV18 positive	43 (0.5)	14 (3.3)	4 (2.5)	1 (2.1)	3 (4.8)	1 (3.4)	23 (3.2)
HPV18 single infection	30 (0.3)	9 (2.1)	3 (1.9)	1 (2.1)	2 (3.2)	1 (3.4)	16 (2.2)
HPV18 multiple infection ^b^	13 (0.1)	5 (1.2)	1 (0.6)	0 (0.0)	1 (1.6)	0 (0.0)	7 (1.0)
Other hrHPV infection	583 (6.2)	174 (41.3)	92 (58.2)	21 (44.7)	29 (46.8)	6 (20.7)	322 (44.9)

Abbreviations: AGC, atypical glandular cells; ASC-H, atypical squamous cells, cannot exclude high-grade lesion; ASCUS, atypical squamous cells of undetermined significance; CIN, cervical intraepithelial neoplasia; HSIL, high-grade squamous intraepithelial lesion; LSIL, low-grade squamous intraepithelial lesion; NILM, negative for intraepithelial lesion or malignancy.^ a^ HPV16 multiple infection indicates HPV16+/HPV18+, or HPV16+/HPV18+/ Other hrHPV+, or HPV16+/Other hrHPV+; ^b^ HPV18 multiple infection indicates HPV18+/Other hrHPV+.

**Table 4:** HPV types and age by cumulative histology at baseline plus 3-year follow-up

HPV infection	No. (%) of specimens with cumulative histology results				
	≤CIN1	CIN2	CIN3+	No histology	Total
**Overall**					
HPV16/18	156 (1.6)	24 (35.8)	29 (50.9)	18 (5.2)	227 (2.2)
HPV16	98 (1.0)	22 (32.8)	27 (47.4)	14 (4.0)	161 (1.6)
HPV18	58 (0.6)	2 (3.0)	2 (3.5)	4 (1.2)	66 (0.6)
Other hrHPV	521 (5.4)	42 (62.7)	25 (43.9)	317 (91.6)	905 (8.9)
Negative	9,039 (93.0)	1 (1.5)	3 (5.3)	11 (3.2)	9,054 (88.9)
**<30**					
HPV16	8 (0.1)	3 (4.5)	2 (3.5)	0 (0.0)	13 (0.1)
HPV18	2 (0.0)	0 (0.0)	0 (0.0)	0 (0.0)	2 (0.0)
Other hrHPV	34 (0.3)	2 (3.0)	0 (0.0)	16 (4.6)	52 (0.5)
Negative	490 (5.0)	0 (0.0)	1 (1.8)	1 (0.3)	492 (4.8)
**30-39**					
HPV16	52 (0.5)	10 (14.9)	11 (19.3)	7 (2.0)	80 (0.8)
HPV18	26 (0.3)	0 (0.0)	1 (1.8)	1 (0.3)	28 (0.3)
Other hrHPV	204 (2.1)	19 (28.4)	11 (19.3)	126 (36.4)	360 (3.5)
Negative	3,856 (39.7)	0 (0.0)	1 (1.8)	5 (1.4)	3,862 (37.9)
**40-49**					
HPV16	22 (0.2)	7 (10.4)	7 (12.3)	6 (1.7)	42 (0.4)
HPV18	19 (0.2)	2 (3.0)	0 (0.0)	2 (0.6)	23 (0.2)
Other hrHPV	217 (2.2)	15 (22.4)	9 (15.8)	113 (32.7)	354 (3.5)
Negative	3,470 (35.7)	1 (1.5)	1 (1.8)	3 (0.9)	3,475 (34.1)
**≥50**					
HPV16	16 (0.2)	2 (3.0)	7 (12.3)	1 (0.3)	26 (0.3)
HPV18	11 (0.1)	0 (0.0)	1 (1.8)	1 (0.3)	13 (0.1)
Other hrHPV	66 (0.7)	6 (9.0)	5 (8.8)	62 (17.9)	139 (1.4)
Negative	1,223 (12.6)	0 (0.0)	0 (0.0)	2 (0.6)	1,225 (12.0)

Abbreviations: CIN, cervical intraepithelial neoplasia.

**Table 5:** HPV types and cytology categories by cumulative histology at baseline plus 3-year follow-up

HPV types and cytology grades	No. (%) of samples with cumulative baseline plus 3-year histological result				
	≤ CIN1	CIN2	CIN3+	No histology	Total
**NILM**					
HPV16	60 (74.1)	8 (9.9)	3 (3.7)	10 (12.3)	81
HPV18	39 (90.7)	1 (2.3)	0 (0.0)	3 (7.0)	43
Other hrHPV	277 (47.5)	7 (1.2)	2 (0.3)	297 (50.9)	583
Negative	8,762 (100.0)	0 (0.0)	0 (0.0)	0 (0.0)	8,762
Subtotal	9,138 (96.5)	16 (0.2)	5 (0.1)	310 (3.3)	9,469
**ASCUS**					
HPV16	24 (75.0)	5 (15.6)	3 (9.4)	0 (0.0)	32
HPV18	13 (92.9)	1 (7.1)	0 (0.0)	0 (0.0)	14
Other hrHPV	152 (87.4)	11 (6.3)	3 (1.7)	8 (4.6)	174
Negative	201 (100.0)	0 (0.0)	0 (0.0)	0 (0.0)	201
Subtotal	390 (92.6)	17 (4.0)	6 (1.4)	8 (1.9)	421
**LSIL**					
HPV16	8 (66.7)	2 (16.7)	1 (8.3)	1 (8.3)	12
HPV18	4 (100.0)	0 (0.0)	0 (0.0)	0 (0.0)	4
Other hrHPV	71 (76.3)	10 (10.8)	3 (3.2)	8 (9.7)	92
Negative	46 (92.0)	0 (0.0)	0 (0.0)	4 (8.0)	50
Subtotal	129 (81.1)	12 (7.5)	4 (2.5)	13 (8.8)	158
**>LSIL^ a^**					
HPV16	6 (16.7)	7 (19.4)	20 (55.6)	3 (8.3)	36
HPV18	2 (40.0)	0 (0.0)	2 (40.0)	1 (20.0)	5
Other hrHPV	21 (37.5)	14 (25.0)	17 (30.4)	4 (7.1)	56
Negative	30 (73.2)	1 (2.4)	3 (7.3)	7 (17.1)	41
Subtotal	59 (42.8)	22 (15.9)	42 (30.4)	15 (10.9)	138
Total	9,716 (95.4)	67 (0.7)	57 (0.6)	346 (3.4)	10,186

Abbreviations: ASCUS, atypical squamous cells of undetermined significance; CIN, cervical intraepithelial neoplasia; hrHPV, high-risk human papillomavirus; LSIL, low-grade squamous intraepithelial lesion; NILM, negative for intraepithelial lesions or malignancy. ^a^ >LSIL indicates atypical glandular cells (AGC), atypical squamous cells, cannot rule out high-grade lesion (ASC-H), and high grade squamous intraepithelial lesion (HSIL).

**Table 6:** The crude risk (%) of each cotesting result for CIN2+/CIN3+ detected at baseline and that detected at baseline and 3-year follow-up

HPV types and Cytology grades	Diseases detected at Baseline				Diseases detected at baseline^ b^ and 3-year follow-up			
	CIN2+		CIN3+		CIN2+		CIN3+	
	n/total	Risk (95%CI)	n/total	Risk (95% CI)	n/total	Risk (95% CI)	n/total	Risk (95% CI)
**HPV16**								
NILM	11/71	15.5 (7.0-23.9)	3/71	4.2 (1.4-8.5)	11/71	15.5 (7.0-23.9)	3/71	4.2 (1.4-8.5)
ASCUS	8/32	25.0 (12.5-40.6)	3/32	9.4 (3.1-19.4)	8/32	25.0 (12.5-40.6)	3/32	9.4 (3.1-19.4)
LSIL	3/11	27.3 (9.1-54.5)	1/11	9.1 (9.1-27.3)	3/11	27.3 (9.1-54.5)	1/11	9.1 (9.1-27.3)
>LSIL ^a^	27/33	81.8 (66.7-93.9)	20/33	60.6 (45.5-75.8)	27/33	81.8 (66.7-93.9)	20/33	60.6 (45.5-75.8)
Subtotal	49/147	33.3 (25.9-40.8)	27/147	18.4 (12.2-24.5)	49/147	33.3 (25.9-40.8)	27/147	18.4 (12.2-24.5)
**HPV18**								
NILM	1/40	2.5 (2.5-10.0)	0/40	NA	1/40	2.5 (2.5-10.0)	0/40	NA
ASCUS	1/14	7.1 (7.1-21.4)	0/14	NA	1/14	7.1 (7.1-21.4)	0/14	NA
LSIL	0/4	NA	0/4	NA	0/4	NA	0/4	NA
>LSIL ^a^	2/4	50.0 (25-75)	2/4	50.0 (25-75)	2/4	50.0 (25-75)	2/4	50.0 (25-75)
Subtotal	4/62	6.5 (1.6-12.9)	2/62	3.2 (1.6-8.1)	4/62	6.5 (1.6-12.9)	2/62	3.2 (1.6-8.1)
**Other hrHPV**								
NILM	2/163	1.2 (0.6-3.7)	0/163	NA	9/286	3.1 (1.0-5.2)	2/286	0.7(0.3-2.1)
ASCUS	12/163	7.4 (3.7-11.7)	1/163	0.6 (0.6-1.8)	14/166	8.4 (4.2-12.7)	3/166	1.8 (0.6-4.2)
LSIL	11/82	13.4 (6.1-20.7)	2/82	2.4 (1.2-6.1)	13/84	15.5 (8.3-22.6)	3/84	3.6 (1.2-8.3)
>LSIL ^a^	30/51	58.8 (45.1-72.5)	16/51	31.4 (19.6-45.1)	31/52	59.6 (44.2-73.1)	17/52	32.7 (21.2-46.2)
Subtotal	55/459	12.0 (9.2-14.8)	19/459	4.1 (2.4-6.1)	67/588	11.4 (9.0-14.5)	25/588	4.3 (2.7-6.0)
**Negative**								
NILM	0/8,762	NA	0/8,762	NA	0/8,762	NA	0/8,762	NA
ASCUS	0/201	NA	0/201	NA	0/201	NA	0/201	NA
LSIL	0/46	NA	0/46	NA	0/46	NA	0/46	NA
>LSIL ^a^	4/34	11.8 (2.9-23.5)	3/34	8.8 (2.9-20.6)	4/34	11.8 (2.9-23.5)	3/34	8.8 (2.9-20.6)
Subtotal	4/9,043	0.0 (0.0-0.1)	3/9,043	0.0 (0.0-0.1)	4/9043	0.0 (0.0-0.1)	3/9,043	0.0 (0.0-0.1)
Total	112/ 9,711	-	51/9,711	-	124/9,840	-	57/9,840	-

Abbreviations: AGC, atypical glandular cells; ASC-H, atypical squamous cells, cannot rule out high-grade lesion; ASCUS, atypical squamous cells of undetermined significance; CIN, cervical intraepithelial neoplasia; hrHPV, high-risk human papillomavirus; HSIL, high grade squamous intraepithelial lesion; LSIL, low-grade squamous intraepithelial lesion; NA, not available. NILM, negative for intraepithelial lesions or malignancy. ^a^ >LSIL indicates AGC, ASC-H, and HSIL. ^b^ within 12 months of the referral to colposcopy after screened tests.

## Abbreviations

AGC: atypical glandular cell
ASC-H: atypical squamous cells cannot exclude high-grade lesion
ASCUS: atypical squamous cells of undetermined significance
CI: confidence interval
CIN: cervical intraepithelial neoplasia
CIN2+/CIN3+: cervical intraepithelial neoplasia grade 2/3 or worse
hrHPV: high-risk human papillomavirus
HSIL: high-grade intraepithelial lesion
LBC: liquid-based cytology
LSIL: low-grade intraepithelial lesion
PPV: positive predictive value
SD: standard deviation
